# AlphaFold model quality self‐assessment improvement via deep graph learning

**DOI:** 10.1002/pro.70274

**Published:** 2025-08-18

**Authors:** Jacob Verburgt, Zicong Zhang, Daisuke Kihara

**Affiliations:** ^1^ Department of Biological Sciences Purdue University Indiana USA; ^2^ Rosen Center for Advanced Computing Purdue University Indiana USA; ^3^ Department of Computer Science Purdue University Indiana USA

**Keywords:** AlphaFold, deep learning, model quality assessment, protein structure prediction

## Abstract

In recent years, significant advancements have been made in deep learning‐based computational modeling of proteins, with DeepMind's AlphaFold2 standing out as a landmark achievement. These computationally modeled protein structures not only provide atomic coordinates but also include self‐confidence metrics to assess the relative quality of the modeling, either for individual residues or the entire protein. However, these self‐confidence scores are not always reliable; for instance, poorly modeled regions of a protein may sometimes be assigned high confidence. To address this limitation, we introduce Equivariant Quality Assessment Folding (EQAFold), an enhanced framework that refines the Local Distance Difference Test prediction head of AlphaFold to generate more accurate self‐confidence scores. Our results demonstrate that EQAFold outperforms the standard AlphaFold architecture and recent model quality assessment protocols in providing more reliable confidence metrics. Source code for EQAFold is available at https://github.com/kiharalab/EQAFold_public.

## 1 INTRODUCTION

With the introduction of AlphaFold2 (AF2) (Jumper et al., 2021) in the Critical Assessment of Structure Prediction (CASP14) (Lensink et al., 2021) protein structure prediction assessment, the field of protein structure prediction rapidly matured, and a copious amount of computationally predicted protein structures became available through online databases such as the AlphaFold Protein Structure Database (AFDB) (Varadi et al., 2022), which supplements experimentally determined structures available at the Protein Data Bank (PDB) (Berman et al., 2000). Along with 3D coordinates of the atoms within each of these predicted structures, each residue is also annotated with a self‐confidence score, which is used to gauge how trustworthy the modeling of that region of the structure is. Within the context of deep learning‐based methods, these confidence scores are often predictions of traditional quality metrics that have been used in the field of protein structure prediction for many years, with the underlying prediction network being trained in conjunction with the structure prediction itself. The primary confidence metric predicted within AF2 is the Local Distance Difference Test (LDDT) (Mariani et al., 2013), which is predicted for each residue. These confidence scores are often critical for downstream applications and researchers to gauge the reliability of structures. Furthermore, in the event that multiple structure predictions exist, as they often do, these confidence scores are used to rank and select them, bolstering the significance of the self‐confidence scores for downstream research.

In a similar vein, there are a number of model quality assessment (MQA) methods (Kihara et al., 2009) that have been developed to assign quality scores to already predicted protein structures (Baldassarre et al., 2021; Olechnovič & Venclovas, 2017; Roche et al., 2023; Roy & Ben‐Hur, 2023; Shin et al., 2017). Instead of relying on self‐confidence metrics generated alongside the predicted models, these methods analyze the resultant protein structures to assign their own confidence scores. This allows for the analysis of protein structures generated from a variety of methods, which is often of use for the CASP Estimation of Model Accuracy category (Kwon et al., 2021), in which participants attempt to rank and select well‐modeled structures from a pool of candidate structures. The graph‐like nature of the LDDT metric has led many of these methods to be graph‐based (Baldassarre et al., 2021; Roche et al., 2023; Roy & Ben‐Hur, 2023).

In this study, we aim to increase the accuracy of AF2 self‐confidence scores through the incorporation of equivariant graph neural networks (EGNNs) (Satorras et al., 2022) in place of the standard LDDT prediction head present within AF2. EGNN architectures are able to leverage relative spatial information within the graph and are widely used for parsing and interpretation of molecular data (Masters et al., 2023; Roche et al., 2023; Wu et al., 2023), including MQA methods (Chen et al., 2023), and have been shown to outperform traditional graph methods in similar tasks (Roche et al., 2023). Titled Equivariant Quality Assessment Folding (EQAFold), this work reimplements and fine‐tunes the LDDT prediction head of a pre‐trained AF2 model to provide more accurate self‐confidence scores. This process differs substantially from standard MQA methods by instead improving the underlying self‐confidence scores that are generated alongside the predicted structure, as opposed to applying an external program for analysis. This provides us with the unique benefit of leveraging the same information that was used to generate the predicted protein structure. We believe that improvement in self‐confidence score accuracy can be achieved through several key concepts: First, the EGNN architecture will allow better predicted Local Distance Difference Test (pLDDT) assignment over the multi‐layer perceptron network used within AF2. In particular, the AlphaFold pLDDT prediction head does not leverage pairwise information, which we will leverage as edges, as well as edge features within our network. Second, in our training dataset, we were careful to exclude training data entries in which the polypeptide chain was extracted from a larger multimeric structure and thus cannot be accurately evaluated as a monomer. Third, we incorporated additional features of a query structure model in EQAFold. In particular, we utilize fluctuations that occur when the model is run multiple times and embedding data from protein language models (Lin et al., 2023; Rives et al., 2021). We benchmarked EQAFold against the standard AlphaFold architecture on a test dataset of 726 monomeric protein structure entries and were able to show that EQAFold can provide more accurate confidence metrics, particularly in regions with substantial LDDT prediction errors.

## 2 RESULTS

### 2.1 The overview of EQAFold

**FIGURE 1:**
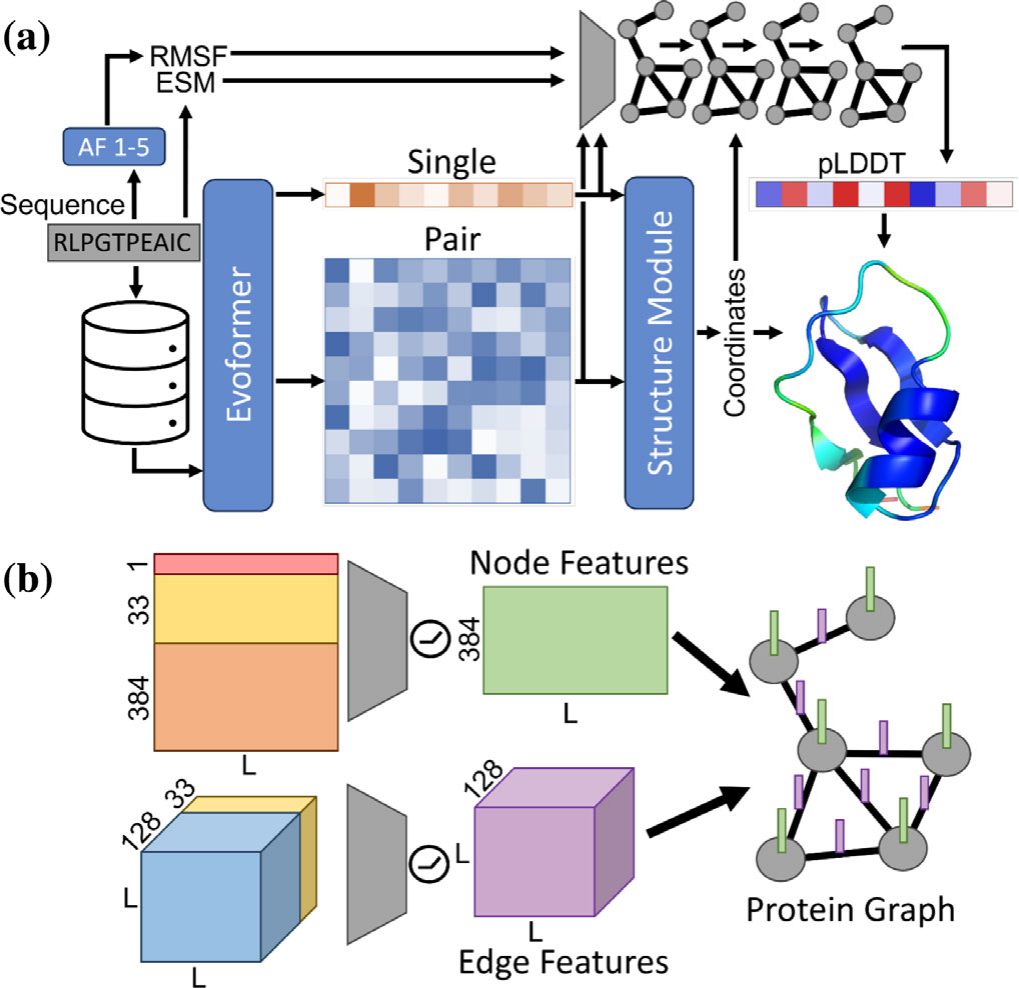
Overview of Equivariant Quality Assessment Folding (EQAFold). (a) The EQAFold architecture largely follows that of AlphaFold2 (AF2). The Local Distance Difference Test (LDDT) prediction head of EQAFold is based on equivariant graph neural networks (EGNNs) that use the predicted Cα coordinates from the structure module to create a graph representation of the protein model. The head leverages information from the single and pair representations computed by AF2, the evolutionary scale modeling (ESM) protein language model, and local variability of five structure models from AF2 in the form of root mean square fluctuation (RMSF). (b) Construction of the Protein Graph Representation for the LDDT Prediction Head. The final single representation (orange), layer‐wise ESM embeddings (yellow), and RMSF values (red) are concatenated and passed through a single linear transition layer with Rectified Linear Unit (ReLU) activation and are subsequently used as node features. AlphaFold pairwise embeddings (blue) and ESM attentions (yellow) are passed through a single transition layer with ReLU activation, contain information for all residue pairs, which are extracted as edge features. The graph representation of the protein is built using Cα distances and has the constructed node and edge features loaded on.

EQAFold adopts the same architecture for structure prediction as AF2 but incorporates an enhanced LDDT prediction head based on EGNNs (Figure 1a). Starting from the input sequence of a target protein, a Multiple Sequence Alignment (MSA) is created through a sequence database search. The MSA is then processed by the Evoformer module, which produces single and pair representations. These representations are subsequently used by the structure module to predict the protein structure. Up to this stage, the data is processed with the AF2 network.

The newly developed LDDT prediction head converts the final single and pair representations from the structure module, the predicted Cα coordinates, layer‐wise embeddings of the ESM2 protein language model (Rives et al., 2021), and the root mean square fluctuation (RMSF) of five structure models (generated via the standard AF2 with 50% dropout in the structure module) into a graph representation of the protein structure. We used RMSF from the five dropout replicates because structural differences among different models have been used in past consensus‐based quality assessment methods (Adamczak et al., 2011; Wang et al., 2011, 2020). As shown in Figure S1, it is clear that RMSF negatively correlates with LDDT, since as AF2 has less information on a residue's position relative to others, its placement in space will vary more.

In the graph (Figure 1b), nodes represent amino acids, while edges are constructed for residues in which Cα positions were within 16 Å. Node features are constructed by concatenating the final single representation from the Evoformer (the dimension is *L* × 384, where *L* is the length of the protein), the averaged ESM layers (*L* × 33), and the RMSF values (*L* × 1) and passing through a linear transition layer with ReLU activations, resulting in *L* × 384 node features (Figure 1b). Edge features are constructed using the pair embeddings of residue pairs (*L* × *L* × 128) and averaged attention layers from ESM (*L* × *L* × 33), as performed previously (Roche et al., 2023). The corresponding residue pair for any edge is extracted and used as edge features. The EGNN‐based prediction network itself consists of four equivariant graph convolutional layers with 384 node features at the input, 128 hidden node features between layers, and 50 output node features.

We primarily base our method off of our in‐house implementation of AF2, which was built on a composite of OpenFold (Ahdritz et al., 2023) and our own newly generated code. Our method starts at the structure module stage of AF2, with the single and pair representations being precomputed via the DeepMind AF2 implementation.

Our training and testing datasets were constructed from the PISCES protein sequence culling server (Wang & Dunbrack Jr., 2003) (see Section 4). For either dataset, we included protein structures that were solved in a monomeric state and had a resolution of at least 2.5 Å. To prevent redundancy between the training and testing data, we ensured that there was no more than 40% sequence similarity between the training and testing data following the criteria used in the AF2 paper. The training dataset contained a total of 11,966 entries, and the testing dataset contained a total of 726 entries. Out of the 726 targets in the test set, 530 of them have corresponding AFDB structures with identical sequences. Cases in which the AFDB structure cannot be acquired are due to a lack of a UniProt ID, vary from the PDB entry, or simply lack an AFDB entry.

### 2.2 Overall results

**FIGURE 2:**
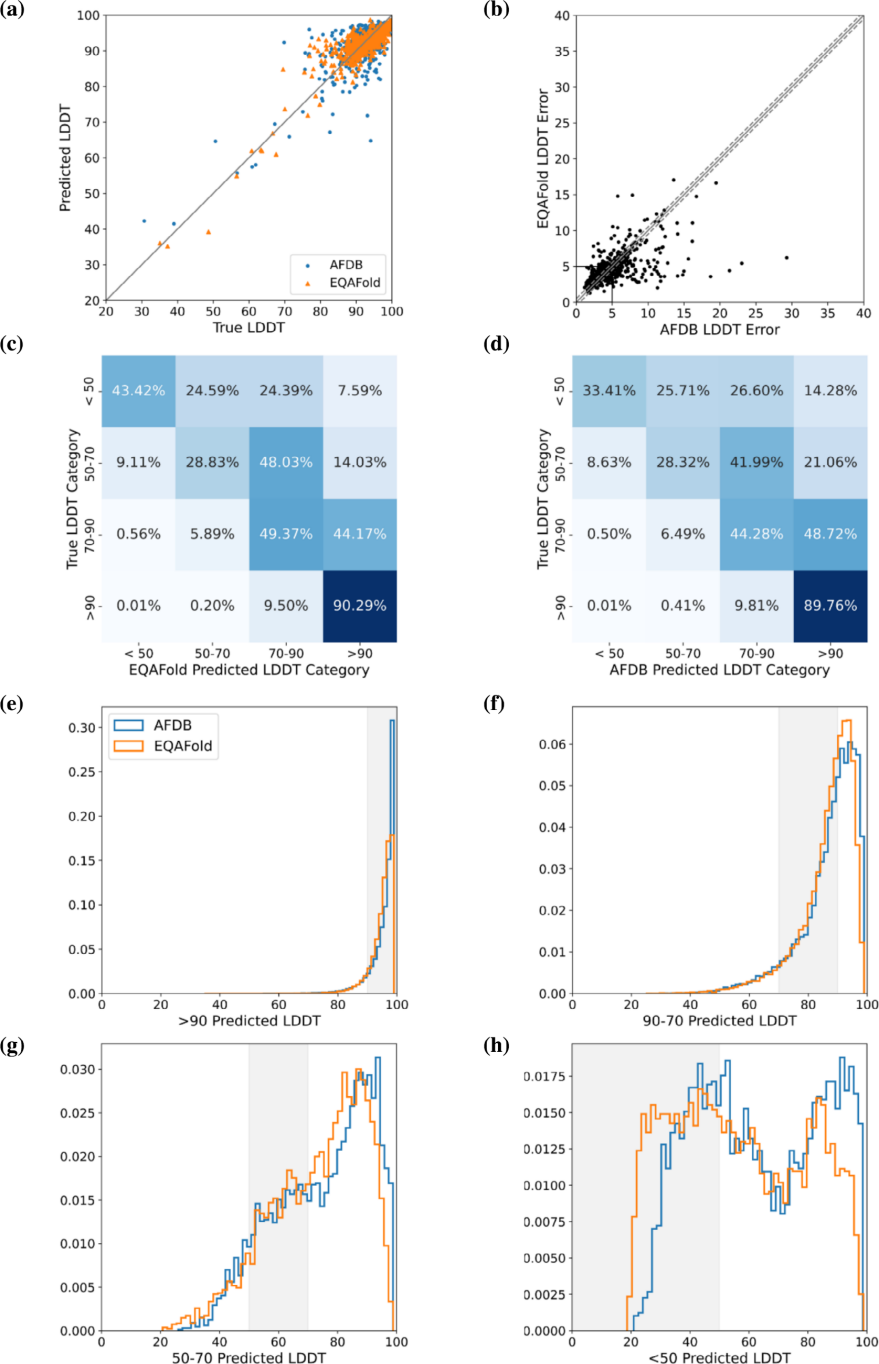
Model quality assignment by Equivariant Quality Assessment Folding (EQAFold). (a) Model‐level pLDDT assignment by AlphaFold Protein Structure Database (AFDB) (blue circles) and EQAFold (orange triangles) relative to the true Local Distance Difference Test (LDDT). The 530 test entries for both EQAFold and AFDB were plotted. (b) Comparison of model‐level LDDT error between EQAFold and AFDB on the 530 test entries. For each model, the error is calculated as the average absolute LDDT error across all residues. The respective errors from EQAFold and AFDB are plotted, with dashed lines indicating a 0.5 LDDT margin, within which differences between the two methods are considered marginal (a tie). (c and d) Residue‐level confusion matrices of EQAFold and AFDB, respectively. For each of the four categories of true LDDT, >90, 90–70, 70–50, and <50, the fraction of predicted LDDT values in each of the categories is shown. (e–h) Residue‐level histograms of the predicted LDDT distributions for both EQAFold and AFDB for each of the four true LDDT categories. The *y*‐axis shows the fraction of residues. The pLDDT range that would be considered correct for each LDDT category is shown in gray.

We analyzed model‐level and residue‐level pLDDT by EQAFold in comparison with AFDB in Figure 2. A model‐level pLDDT is the average pLDDT of all the residues in a protein model. First, in Figure 2a, we compared the model‐level pLDDT of the two methods with the true LDDT. Overall, both methods predicted LDDT values close to the true LDDT for the majority of the targets. For 348 (65.7%) and 316 (59.6%) of the targets, EQAFold and AFDB, respectively, predicted LDDT within a margin of 0.5 LDDT error. The average pLDDT errors of EQAFold and AFDB were 4.74 and 5.16, respectively. EQAFold and AFDB performed similarly in terms of structure model accuracy. The difference in their average LDDT scores was 0.84, and for 72.6% of the targets, the LDDT values of the two methods differed by less than 0.5 (Figure S2).

However, differences between the two methods become apparent when analyzing pLDDT errors. Figure 2b presents the pLDDT error, defined as the difference between pLDDT and the true LDDT, for EQAFold and AFDB. By analyzing the pLDDT error instead of absolute pLDDT values, we largely avoid biases from deviations in the underlying structures from AFDB and EQAFold. Since our focus is on models with significant LDDT errors, we examined the subset where either method had an average LDDT error greater than 5.0. Considering 256 targets where either method had a significant LDDT error (average LDDT error of above 5.0), EQAFold had lower LDDT error than the deposited AFDB model in 127 (49.61%) of the targets, with 56 (21.88%) of the models being tied, where the difference of errors of the two methods was less than 0.5. Only 73 (28.52%) of AFDB models with substantial modeling error had lower LDDT error than EQAFold.

We further investigated the residue‐level accuracy of EQAFold versus AFDB in Figure 2c,d. We binned the predicted and true LDDT values for each residue into the four categories utilized in the AFDB: >90, 70–90, 50–70, and <50. We compare the correct prediction of LDDT category by both EQAFold (Figure 2c) and AFDB (Figure 2d). The difference between EQAFold and AFDB becomes more apparent when we consider the misclassification of residues by two or more categories. For instance, among 3977 residues with a true LDDT <50, EQAFold assigned a high pLDDT (>90) in 7.59% of cases, whereas AFDB made twice as many such errors (14.28%). Similarly, for residues with a true LDDT of 50–70, EQAFold assigned a high pLDDT in 14.03% of cases, while AFDB did so 50% more frequently (21.06%). Such overestimation poses risks for practical applications of AF models, as users may mistakenly trust and utilize inaccurately modeled structures when pLDDT values are high. In Figure 2e–h, the distribution of predicted LDDT values for the four true LDDT categories is shown. For the >90 true LDDT category (Figure 2e), although pLDDT by AFDB is skewed toward very high values, EQAFold has a higher fraction predicting LDDT in the correct >90 categories, 90.29% (Figure 2c), as opposed to 89.76% by AFDB (Figure 2d). This difference in the correct fraction turned out to be statistically significant by a chi‐squared test (*p* = 1.71e−5). The 70–90 true LDDT category (Figure 2f) showed that EQAFold again increased the proportion of correctly categorized residues significantly compared to AFDB (44.28–49.37) (*p* = 8.43e−27). The 50–70 true LDDT category (Figure 2g) did not show any statistically significant improvement using a *p*‐value cutoff of 0.05 (*p* = 0.59), despite EQAFold correctly categorizing slightly more residues than AFDB. In the <50 true LDDT category (Figure 2h), EQAFold substantially increased the proportion of correctly categorized residues over AFDB (33.41%–43.42%) (*p* = 1.46e−17).

### 2.3 Examples of EQAFold's prediction

**FIGURE 3:**
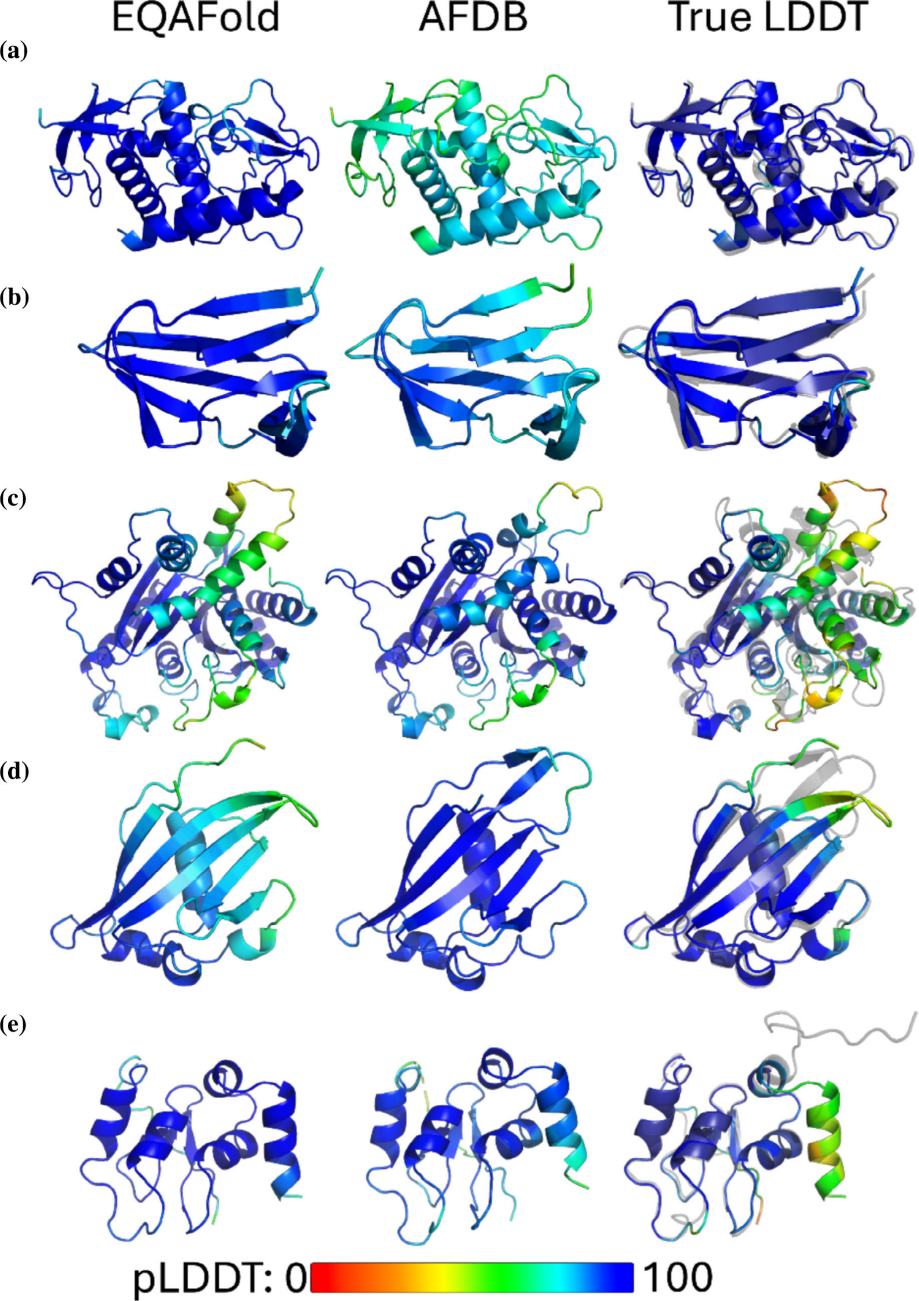
Examples of predicted Local Distance Difference Test (LDDT) by Equivariant Quality Assessment Folding (EQAFold) and the AlphaFold Protein Structure Database. Per‐residue pLDDT is shown for EQAFold, and the AlphaFold Protein Structure Database (AFDB) deposited structure. Colors represent the pLDDT value, which ranges from 0 (red) to 100 (blue). The true LDDT for the predicted structure of EQAFold superimposed with the experimental structure (gray) are also shown in the third column. (a) HORMA DNA binding domain‐containing protein (Protein Data Bank [PDB]: 7UBA, UniProt ID: A7TRP1). (b) DNA‐directed RNA polymerase subunit beta (PDB: 8H02, UniProt ID: Q31N15). (c) Human guanylate‐binding protein 2 (PDB: 6VKJ, UniProt ID: P32456). (d) Ubiquitin‐activating enzyme E1‐like (PDB: 3ONH, UniProt ID: P52488). (e) Human BIR2 domain of BIRC1 (2VM5, UniProt ID: P52488).

In Figure 3 we show several examples of pLDDT by EQAFold in comparison with AFDB. The first example (Figure 3a) is a 208‐residue long α‐helical protein. The predicted models by EQAFold and AFDB for this target were highly accurate with RMSDs of 0.65 and 0.67 Å, respectively. The correct LDDT of residues in these models are within the range of 76.72–100, with an average LDDT of 98.18, as shown in blue in the true LDDT panel. EQAFold successfully recognized the correctness of the model with pLDDT values in the ranges of 70.51–97.18, with an average pLDDT of 92.76, whereas AFDB underestimated the quality, assigning lower pLDDT values ranging from 47.53 to 83.19, with an average pLDDT of 71.84. Figure 3b is a similar example for a β‐class protein. The models are highly accurate, but AFDB estimated lower quality toward the two ends of the chain. Out of the 79 residues in total, 74 residues have a pLDDT value less than 90 in the AFDB model. Figure 3c shows the LDDT assignment for Human guanylate‐binding protein 2. Both EQAFold and AFDB failed to model a helix and loop in the center of the protein correctly. The RMSDs of the EQAFold and AFDB models were 3.37 and 3.53 Å, respectively. Despite this failure in modeling, AFDB was generally confident in this region, labeling it with predicted LDDT values between 80.61 and 91.35. On the other hand, EQAFold recognized the inaccurate helix and loop region correctly, assigning with LDDT values in the range of 49.00–76.17 (yellow to green in the figure).

The next example in Figure 3d is an opposite case where EQAFold did slightly worse in pLDDT prediction. In this example, the structure model itself by EQAFold had some differences from the corresponding AFDB model. EQAFold has a lower accuracy (LDDT from 40.83 to 70.00) in a loop that connects two β strands (residue 79–87), where LDDT is colored in green in the true LDDT panel. For these residues, EQAFold identified them as being low quality, with pLDDT of 54.56–76.04, but the residue‐level pLDDT error was between 3.42 and 23.57. On the other hand, the AFDB model (middle) is more accurate with LDDT of 96.83 as opposed to 89.05 for the EQAFold model. The residue‐level LDDT of the β‐strand region of the AFDB model was 84.17–97.00, which was predicted to be between 67.35 and 92.31, which was on average 2.63 smaller pLDDT error than EQAFold. Despite the modeling error of EQAFold, it is able to convey the poor modeling of this region such that a researcher would be aware of the quality and avoid misuse of the model.

The last example in Figure 3e is a case where both EQAFold and AFDB were overconfident in a low accuracy region. However, this region is a C‐terminal disordered tail, which was predicted as a stable helix by both methods. True LDDT of this region ranges between 32.5 and 62.5, while pLDDT of EQAFold and AFDB were 72.16–96.52 and 61.38–92.92, respectively.

### 2.4 Feature analysis

**FIGURE 4:**
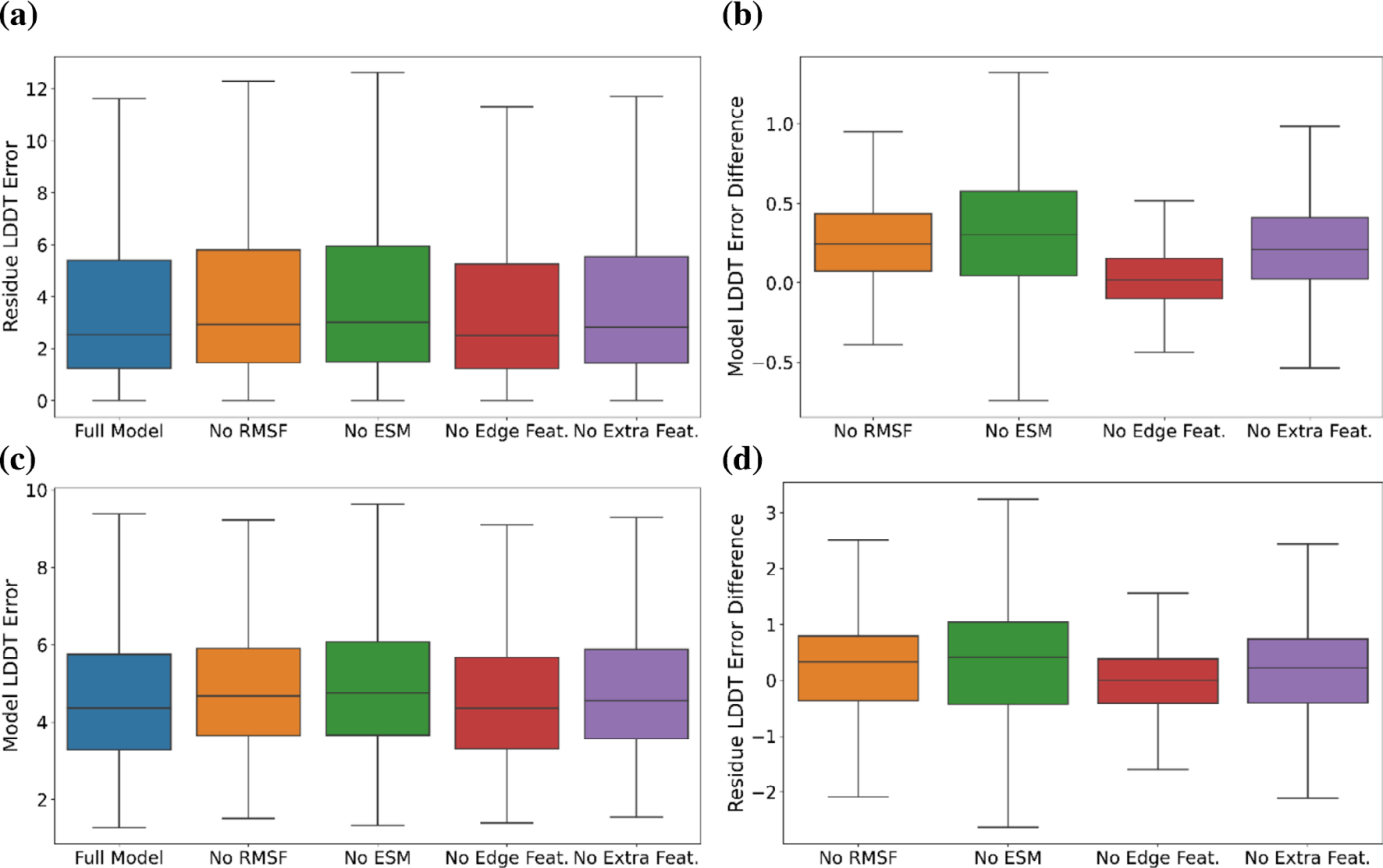
Ablation study of features used in Equivariant Quality Assessment Folding (EQAFold). We compared the relative Local Distance Difference Test (LDDT) errors between the full model and four model variants for all the 726 targets in the test set. (a) Model‐level LDDT errors of the full model as well as the four model variants. Box plots show the 25%, 50%, and 75% quantiles, with whiskers showing the span of remaining distribution within 1.5 times the interquartile range. (b) The model‐level LDDT error difference for each of the four model variants relative to the full model. For each target, the LDDT error of the full model was subtracted from the LDDT error of the model variants. Thus, a positive value indicates that the model variant had a larger LDDT error than the full model. (c) The residue‐level LDDT errors of the full model and the other four model variants. (d) The residue‐level LDDT error difference of the four model variants relative to the full model. RMSF, root mean square fluctuation.

To evaluate the relative contribution of node and edge features, we performed feature analysis by retraining several variant models with certain features, or sets of features omitted (Figure 4). The results shown are on the 726 test targets. These variants include a model without any RMSF features (No RMSF), no ESM features (No ESM), no Edge Features (No Edge), and only the AF single representation (No Extra Features). For each model variant, we compute the LDDT errors, as well as the LDDT error differences on both the model level and residue level.

Figure 4a,b show the model‐level LDDT errors and differences of LDDT errors, relative to the full model. The full model had an average LDDT error of 4.89, and the model variants all had higher average LDDT errors of 4.93, 5.20, 5.14, and 5.14 for No Edge, No ESM, No Extra Features, and No RMSF, respectively. Among the three features, lacking the ESM feature increased the error most, while the edge feature had the least influence when removed.

When looking at the residue‐level pLDDT errors in Figure 4c,d, the full model had an average LDDT error of 4.80, and the model variants all had higher average LDDT errors of 4.80, 5.13, 4.98, and 5.04 for No Edge, No ESM, No Extra Features, and No RMSF, respectively. Comparing the three individual features, consistent with the model‐level pLDDT errors, ESM had the largest impact when removed, and the removal of edge features had the smallest impact. Consistent with the model‐level error, removing features did not show a distinct drop in the LDDT prediction accuracy. In addition, we also compared EQAFold with the EGNN architecture and the multi‐layer perceptron architecture without any additional features. The EGNN architecture showed improvement over the standard Multi‐Layer Perceptron (MLP) architecture (Figure S3).

### 2.5 Comparison with EnQA

**FIGURE 5:**
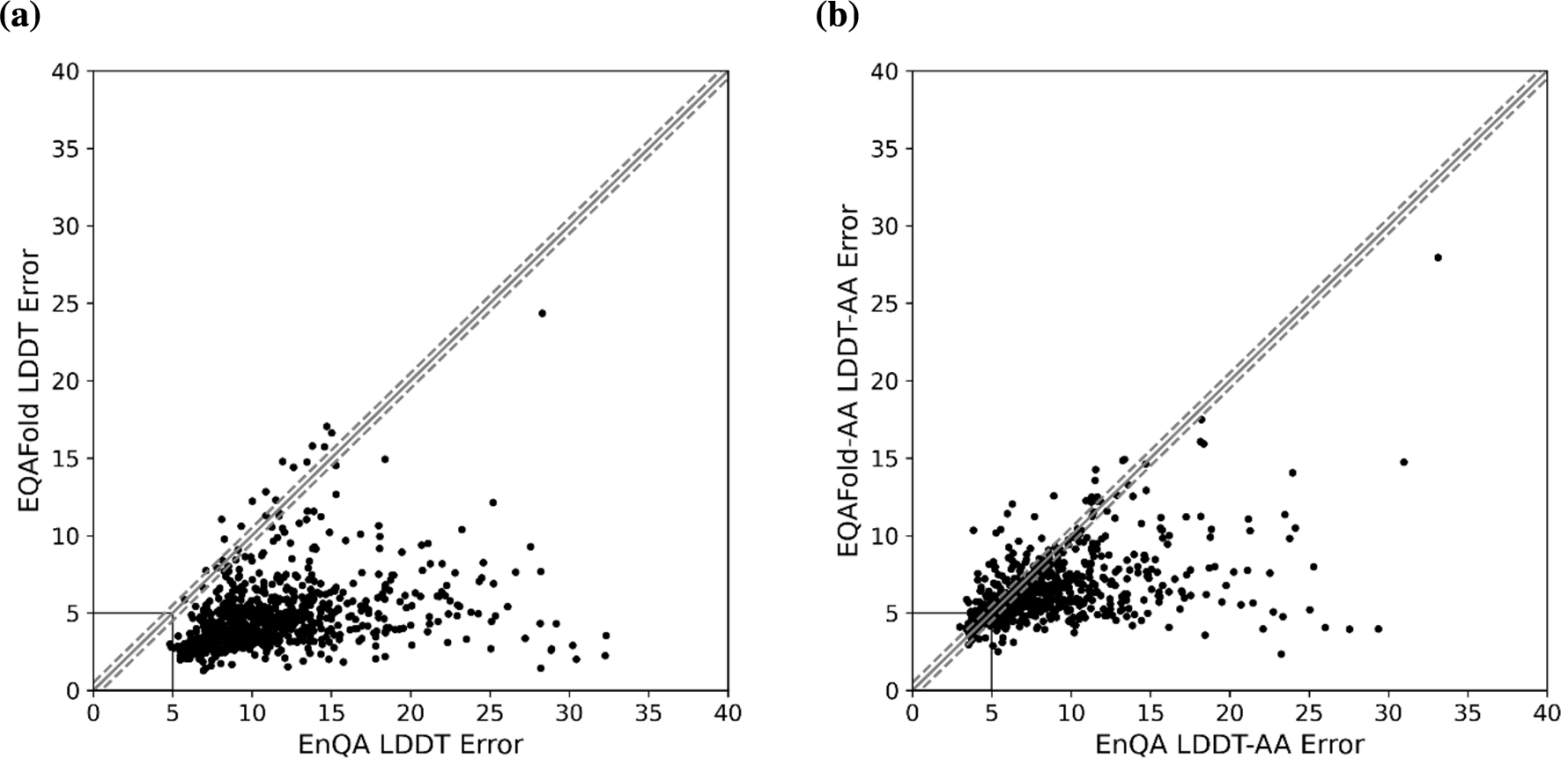
Comparison of Equivariant Quality Assessment Folding (EQAFold) to EnQA. (a) Deviations in average Local Distance Difference Test (LDDT) error between EnQA annotated models and EQAFold on all targets present within the test dataset. The error for any given model is calculated by averaging the absolute LDDT error for any given residue for all residues in the model. Each black point represents a target in the test set. The black box indicates the boundary in which both methods had substantial (>5.0) LDDT error. The solid gray line represents the *y* = *x* line, and dashed gray lines represent the boundary in which LDDT errors are considered equivalent (0.5). (b) Deviations in average all‐atom LDDT (LDDT‐AA) error between EnQA annotated models and EQAFold on all targets present within the test dataset. The error for any given model, the error is calculated by averaging the absolute LDDT‐AA error for any given residue for all residues in the model. Each black point represents a target in the test set. The black box indicates the boundary in which both methods had substantial (>5.0) LDDT error.

Finally, we compared the quality estimation by EQAFold with a recently published method, EnQA (Chen et al., 2023). EnQA takes predicted protein structures as input and utilizes EGNNs and spherical embeddings, using features derived from physical attributes such as the solvent accessible surface area, buriedness, and Voronoi cell volume, as well as ESM2 embeddings to re‐annotate the pLDDT values of the provided structure. We ran the recommended MSA version of EnQA on the 726 models in the test set and compared them with EQAFold. Figure 5a shows errors of LDDT prediction for structure models computed by the two methods. As shown, pLDDT error by EQAFold was substantially smaller than EnQA (i.e., have improvements in LDDT error larger than the 0.5 margin) for the majority (96.7%) of the cases.

While comparing pLDDT values predicted by EQAFold and EnQA, we noticed that EnQA appears to mistakenly be calculating the all‐atom LDDT metric (LDDT‐AA), instead of the standard LDDT metric, which only considers the positions of Cα atoms. LDDT‐AA considers all heavy atoms (not just Cα atoms) within the inclusion radius for any given residue. LDDT‐AA calculates an LDDT value for each atom, from which a residue‐based LDDT can be computed as an average over all atoms in a residue. In practice, this makes the LDDT‐AA metric a more stringent version of the LDDT metric (Figure S4). LDDT‐AA can be useful for discerning small modeling errors in regions where the LDDT metric is nearly perfect. LDDT computation for a protein structure is performed using OpenStructure (Biasini et al., 2013), which allows switching between regular LDDT and LDDT‐AA via an option on the command line. However, the code of EnQA indicates that their training data were generated with the default options, leading to LDDT‐AA being used as the ground‐truth, which may be one of the reasons for the overall larger pLDDT errors by EnQA observed in Figure 5a. This finding prompted us to retrain EQAFold to predict the more stringent LDDT‐AA metric. We facilitate this training by modifying the LDDT‐Loss function to consider all atoms between the true and predicted structures when calculating the true LDDT values. The predicted LDDT‐AA errors by the newly trained EQAFold, named EQAFold‐AA, are plotted against the LDDT‐AA errors of EnQA‐MSA in Figure 5b. Of the 726 targets in the testing set, we find that EQAFold‐AA outperforms EnQA on 414 (57.02%) of targets, with 201 (27.69%) of targets being within the “tied margin” where the difference of the errors by the two methods was within 0.5. Only 111 (15.29%) of models were better modeled by EnQA.

## 3 DISCUSSION

Self‐confidence metrics in protein structure prediction, such as the pLDDT score, play a crucial role in ranking and analyzing computationally predicted protein structures. These metrics are critical for end users, as they guide decisions on whether to trust a predicted model, particularly in applications such as structural biology, drug discovery, and functional annotation. In this work, we demonstrate that pLDDT in AF2 can be further refined using enhanced deep learning strategies. Specifically, the EGNN framework employed in EQAFold, which is explicitly integrated within the AF2 network architecture, enables more precise pLDDT annotations by leveraging geometric and relational information.

As seen in the feature analysis (Figure 4), removing the extra features of ESM embeddings and attentions was the most detrimental to the accuracy of the model. This is likely due to the supplementary information it can provide outside of the single and pair representations. As AF2 single and pair representations are used in both structure prediction and confidence assessment, any underlying errors in these representations that result in poor structure modeling are also likely to result in poor confidence assessment. As the ESM embeddings are not used for structure modeling, they can offer novel additional information to the confidence assessment. Further, unlike the AF2 single and pair representations, ESM embeddings are not dependent on an MSA built from sequence databases but instead result from a generalized protein language model.

As of current, EQAFold, built on the architecture of AF2, is only truly capable of predicting the structures of monomeric proteins. In the current version, due in part to limited computational resources, the pLDDT and structure modules were trained separately while the remaining components of AF2 were kept frozen. End‐to‐end training of the entire model is expected to further improve prediction accuracy. Further, we believe similar approaches could be broadly applicable to various deep learning‐based protein structure prediction tasks. Future expansions include improved accuracy assessment for antibody prediction, protein complexes, peptide‐docking (Zhang et al., 2025), nucleic acids, and small molecules as such modeling has become possible with more recent structure prediction methods (Abramson et al., 2024) and protein design (Watson et al., 2023). By integrating more advanced confidence estimation techniques, the reliability and interpretability of predicted protein structures can be significantly enhanced, benefiting both computational and experimental biologists.

## 4 MATERIALS AND METHODS

### 4.1 Datasets

We derived our datasets from the PISCES protein sequence culling server (Wang & Dunbrack Jr., 2003). Primarily, we use data pulled from the December 14, 2020, release, culled at 90%, determined by x‐ray crystallography, and have a resolution of 2.5 Å or better. These protein chains were fetched from the PDB and renumbered to match their respective FASTA sequence, resulting in a total of 33,635 protein chains. For fine‐tuning the LDDT prediction head, we further filtered our dataset to only contain protein chains where each chain exists as a single protein. The protein stoichiometry was determined by referring to the equivalent biological assembly of the PDB entry of the chain and filtering out models where multiple chains were present within the biological assembly and had a minimum of 40 resolved residues. This resulted in a subset of 11,966 single‐chain proteins.

The primary validation data was obtained from the PDB on February 28th, 2022. The entire PDB was filtered to find chains derived from x‐ray structures with 2.5 Å or higher resolution, *R*_free_ ≤0.25, and contained at least 40 residues but no longer than 1000 residues in the sequence. These sequence chains were then clustered against the previous PISCES training dataset via MMseqs2 (Steinegger & Söding, 2017) with a 25% similarity cutoff. These protein chains were fetched from the PDB and renumbered to match their respective FASTA sequence, resulting in a total of 363 structures after feature generation.

The testing dataset was generated from the May 31st, 2023, release of the PISCES protein sequence culling server with the same constraints. The testing dataset is built to be nonredundant against all previous EQAFold training data to prevent any data leakage that could potentially bias the results toward EQAFold. We first remove entries with identical PDB IDs and subsequently cluster the respective sequences against all chains in the PISCES training dataset and the validation dataset via MMseqs2 (Steinegger & Söding, 2017) with a 40% similarity cutoff. These protein chains were fetched from the PDB and renumbered to match their respective FASTA sequence, resulting in a total of 3582 structures after feature generation. We further filtered our testing dataset to only contain protein chains where each chain exists as a standalone protein by referencing the biological assembly and had a minimum of 40 resolved residues, resulting in a total of 726 single‐chain proteins.

### 4.2 Training procedure

The base network was built and trained with PyTorch 1.12.0, with graph operations being done in PyTorch‐Geometric 2.3.0 and the EGNN implementation from the nascent paper by Satorras et al. (2022) Source code for EQAFold is available at https://github.com/kiharalab/EQAFold_public.

EQAFold was trained in two steps partly due to the limitation of graphics processing unit (GPU) resources we had. First, we trained our in‐house structure module, along with the standard AlphaFold pLDDT prediction head. We needed to train the in‐house structure module by ourselves because, at the time of development, weights from OpenFold were not available and the weights from the AF2 code did not produce reasonable protein structures. After the first stage of training, all structure module parameters were frozen, and the standard LDDT prediction head was replaced by the EGNN model, which was subsequently trained, with all weights within the structure module frozen. This two‐phase training allows us to focus on one objective at a time without inadvertently worsening the quality of the structure generated. But on the other hand, end‐to‐end training would improve the overall accuracy of both structure prediction and confidence prediction. We had examined a couple of cutoff values of contact cutoffs (Yuan et al., 2012) between Cα atoms starting from 8 Å and decided to use 16 Å mainly by visual inspection of edges formed in graphs.

Training was performed on a single Nvidia A100 on our university GPU computer cluster. The structure module was trained with a learning rate of 10^−3^, and the fine tuning of the EGNN prediction head was trained at a rate of 10^−4^.

Due to the high accuracy models in the training dataset skewing the LDDT distribution toward high values, we assigned weights to residues classified into 50 bins, each representing an LDDT range of 2. The weight for an LDDT bin was set to 1 – (the relative fraction of LDDT values that fall within that bin in the training data). In practice, this down‐weights and corrects for the overrepresented LDDT values in the loss calculation.

### 4.3 LDDT computation

The LDDT score provides a superimposition‐free metric to gauge the relative modeling quality of a predicted protein structure to that of an experimentally resolved ground‐truth structure (Mariani et al., 2013). Given a residue in a predicted structure and an experimentally determined ground‐truth structure, the LDDT score looks at all Cα atoms within a 15 Å inclusion proximity within the Cα atom of the residue of interest in the true structure. For each of these Cα atom pairs, the relative distance between the ground‐truth and the predicted structures is compared. Specifically, the score checks if the differences in the distances are within 0.5, 1, 2, and 4 Å of each other. A Cα atom pair can meet all thresholds (resulting in a score of 1) or none of the thresholds (resulting in a score of 0). This is repeated and averaged for all Cα atom pairs within the inclusion proximity around the protein of interest to generate an LDDT score for that residue and is subsequently repeated for all residues within the protein. Alternatively, all heavy atoms of the residue of interest can be considered, resulting in a different and more stringent score. We term this the “all‐atom LDDT” (LDDT‐AA) score. These scores are often scaled between 0 and 100 when annotating predicted structures.

Ground‐truth LDDT values were calculated using the OpenStructure executable (Biasini et al., 2013; Mariani et al., 2013) version 2.3.1. For the standard Cα only LDDT values, the “‐c” flag was used to only consider Cα atoms. To compute all‐atom LDDT values (LDDT‐AA), no flag was used to consider all heavy atoms. We primarily gauge the performance of LDDT assignments by computing the LDDT error for each target in the test dataset. To compute the error, the LDDT error for any given residue *i* is computed by taking the absolute difference between the predicted LDDT value and the true LDDT value (Equation 1). These values are then averaged for all resolved residues in the protein model to compute the LDDT error (Equation 2). Results are visualized in PyMOL version 2.4 (Schrödinger, LLC, 2015).

(1) $$∆{\text{LDDT}}_{i}=\left|{\text{LDDT}}_{i}^{\text{pred}}-{\text{LDDT}}_{i}^{\text{True}}\right|.$$

(2) $${\text{LDDT Error}}_{\text{Model}}=\frac{1}{N}\sum_{i=1}^{N}∆{\text{LDDT}}_{i}.$$

### 4.4 Existing methods

#### 4.4.1 AlphaFold database

For every target within the test dataset, we collect the deposited AlphaFold Protein Structure DataBase (AFDB) (Varadi et al., 2022) model via the web Application Programming Interface (API) by cross‐referencing UniProt IDs (The UniProt Consortium, 2023) between the PDB entry and AFDB, for entries in which a UniProt ID was available and an AFDB entry was present for that ID. We ensured that the AFDB model sequence matches the PDB sequence exactly and removed cases where the sequences deviate. We opt for deposited AFDB models rather than running AF2 by ourselves to ensure that AF2 was run completely to DeepMind specifications. We renumber the AFDB model to match the residue numbering used in the testing dataset. In total, 530 of the 726 test set structures had AFDB entries successfully retrieved and formatted.

#### 4.4.2 EnQA

We also compare against a recent state‐of‐the‐art protein MQA method, which also employs EGNNs, EnQA (Chen et al., 2023). EnQA employs two EGNN networks (node and edge networks) to assign pLDDT values to residues. Within these networks, EnQA primarily utilizes the esm2_t6_8M_UR50D protein language model, spherical embeddings, Voronoi cell volume, solvent accessible surface area, and residue buriedness to generate its pLDDT predictions. For each target in the testing dataset, the predicted structure was passed to EnQA to reassign the pLDDT values for each residue in each target.

## AUTHOR CONTRIBUTIONS

**Jacob Verburgt:** Methodology; software; validation; investigation; formal analysis; visualization; writing – original draft. **Zicong Zhang:** Methodology; software. **Daisuke Kihara:** Conceptualization; investigation; funding acquisition; writing – review and editing; resources; supervision; project administration.

## CONFLICT OF INTEREST STATEMENT

The authors declare no conflict of interest.

## Supporting information


**Data S1.** The supplementary file contains three figures tangential to the dataset and results described in the main text. Figure S1 depicts the correlation of LDDT and RMSD. Figure S2 shows LDDT of AFDB and EQAFold models. Figure S3 shows the relative change in LDDT error with EGNN and MLP architectures in EQAFold. Figure S4 depicts the relationship between LDDT and LDDT‐AA.
