# Supplementary material for: AlphaFold model quality self‐assessment improvement via deep graph learning
Source: Protein Sci. 2025 Aug 18;34(9):e70274. doi: 10.1002/pro.70274 (PMC12359199; doi:10.1002/pro.70274)
Supplement: Supplementary file 1 — Data S1. The supplementary file contains three figures tangential to the dataset and results described in the main text. Figure S1 depicts the correlation of LDDT and RMSD. Figure S2 shows LDDT of AFDB and EQAFold models. Figure S3 shows the relative change in LDDT error with EGNN and MLP architectures in EQAFold. Figure S4 depicts the relationship between LDDT and LDDT‐AA. [file PRO-34-e70274-s001.docx]

**Supplementary Information for**

**AlphaFold model quality self-assessment improvement via deep graph learning**

Jacob Verburgt, Zicong Zhang, and Daisuke Kihara

Purdue University, Department of Biological Sciences, Department of Computer Science

Contact: dkihara@purdue.edu


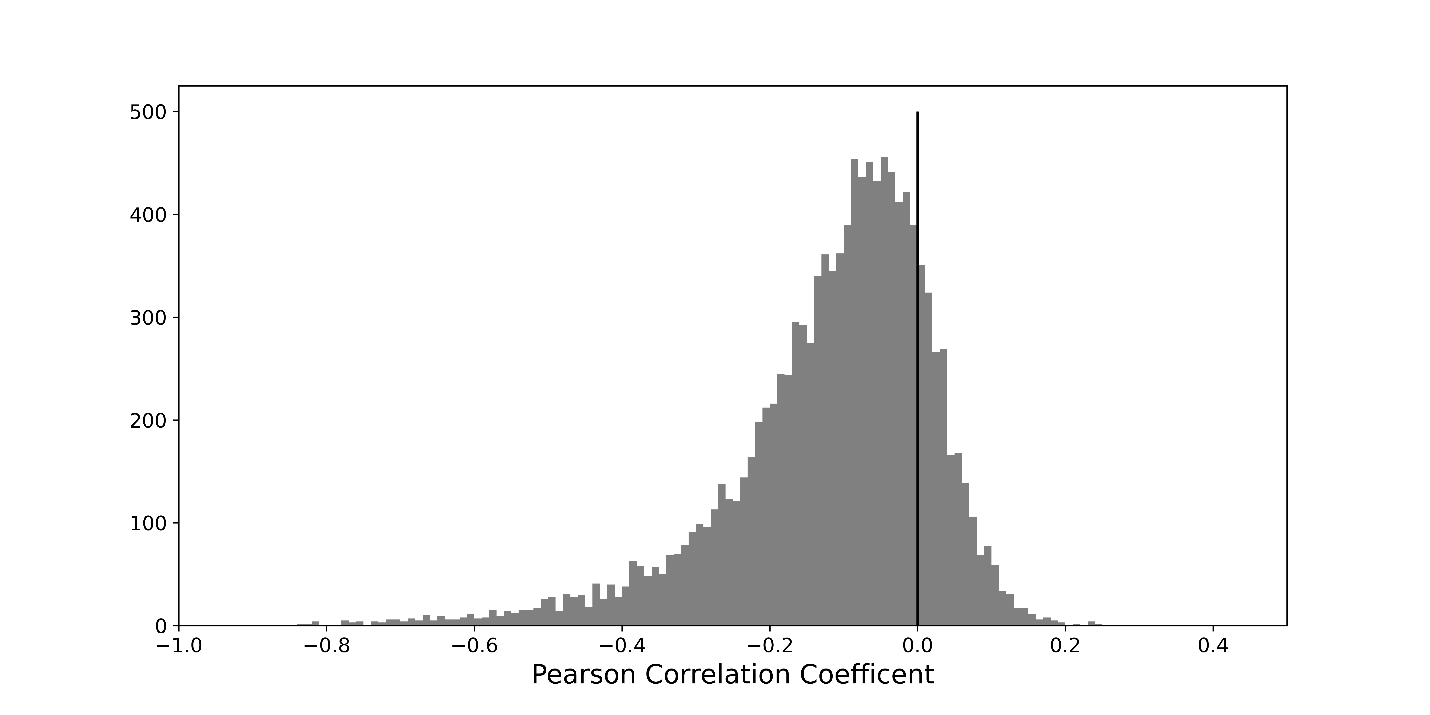


**Supplementary Figure S1: Distribution of Model Level correlations Between RMSF and LDDT.** For each target within the training set, the RMSF value between all 5 dropout replicates was calculated for each residue and are compared to their respective LDDT value. Using all residues within each target, the Pearson correlation was calculated. The black line indicates a correlation coefficient of 0.


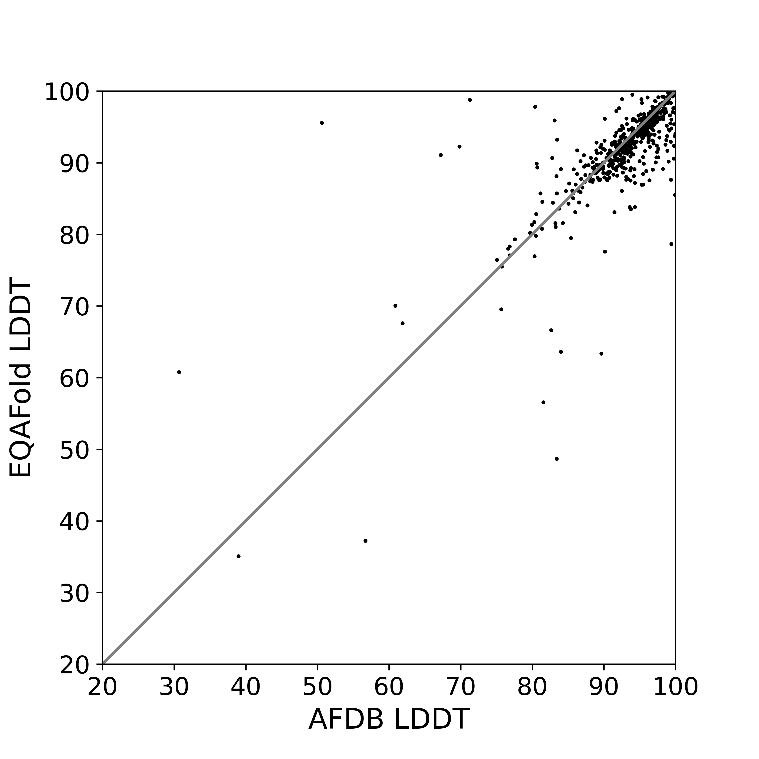


**Supplementary Figure S2: LDDT of models from AFDB and EQAFold.** The average LDDT of the AFDB and EQAFold models were 92.62 and 91.84, respectively (total 530). Using the four LDDT level classifications, >90, 90-70, 70-50, <50, used in the Alphafold Database, 83.96% of the models by AFDB and EQAFold are in the same category, and additional 15.28% are off by one category. Thus 99.24% are within one category difference.


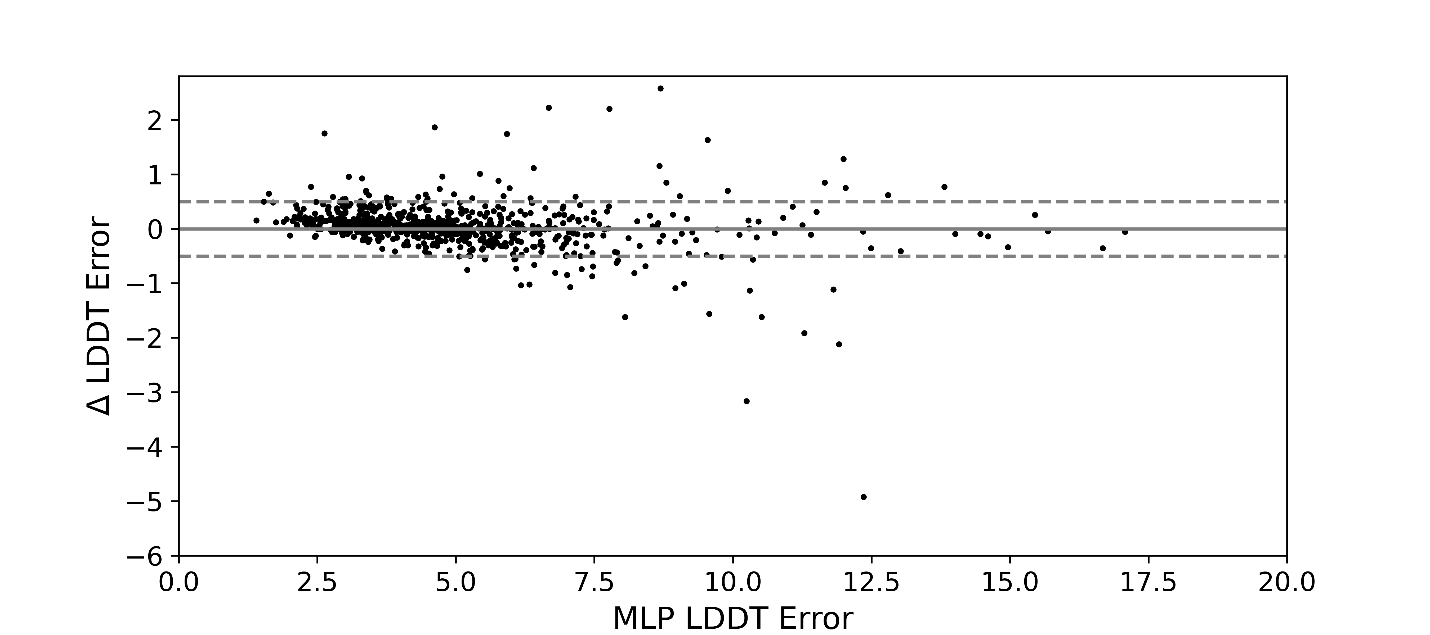


**Supplementary Figure S3: Relative Change in LDDT Error with EGNN and MLP Architectures in EQAFold.** For each target within the testing set, the relative change in model-level LDDT error between the EGNN architecture with no additional features, versus the Multi-Layer Perceptron (MLP) architecture of the AF3, which has 3 hidden layers each of size 128. Dotted lines indicate the margin where LDDT error is considered negligible (within 0.5). The average ∆LDDT Error by the MLP and the EGNN were 5.11 and 5.14, respectively. The number of cases where EGGN had a smaller/larger error (by more than 0.5) was 34 and 45, respectively, and they tied (within a 0.5 difference) for 647 cases.


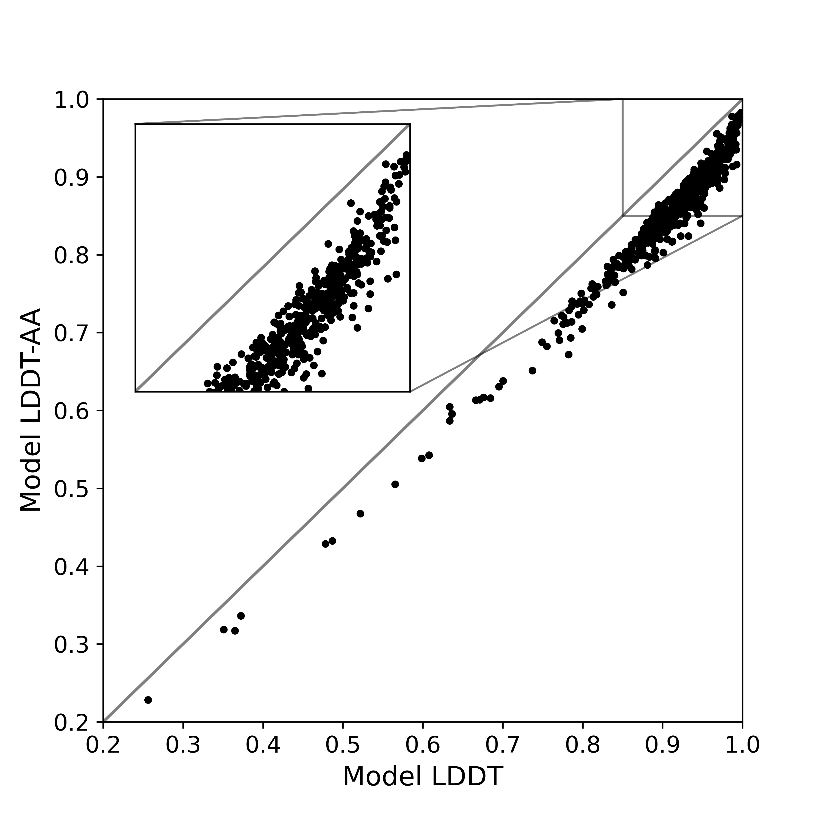


**Supplementary Figure S4: Correlation between LDDT and LDDT-AA.** For each target within the test set, the model level LDDT and LDDT-AA values are plotted against each other.
